# The Dental Office & Laboratory

**Published:** 1877-07

**Authors:** 


### EDITORIAL, ETC.

---

*The Dental Office and Laboratory.*—We are pleased to again welcome a new series of this new dental publication, the second number of which has just been received. The general plan is the same as the first series, and the quarterly numbers contain some of the best of original and selected articles.

It is published as before by Johnson & Lund, of Philadelphia. The following extract is from an article by Professor Buckingham, on Advertising:

"The Dentists, so far as advertising is concerned, may be classed with the Medical profession, but in their intercourse with their patients they are usually much more communicative, and are willing to inform them of not only what is needed to be done, but the best manner of doing it. Advertising, like all other good things, may be abused ; as when the merchant condescends to extol his goods above his neighbors : or say he can sell the same class of goods much cheaper than they can be bought elsewhere, and intimates that if they sell them at the prices he does, they must sell inferior goods, as he has facilities for obtaining his stock that no others have ; or when a manufacturer happens to come across the goods of another manufacturer, which are more or less imperfect, and compares them with some of the best of his own, magnifying the imperfections on one side and the perfections on the other, and publishing them as inducements to draw customers. It can hardly be said that they are doing to others as they would like others to do unto them, and if they treat those in the same business in the manner indicated they are likely to treat their customers worse ; for we may lay it down as a rule that a person who will condescend to resort to treachery to obtain customers, will not hesitate to deceive them when they deal with him. Some think if they can push clear of the law, they are justified in doing anything. This class usually find their position in society sooner or later. The lawyer who takes advantage of all the quirks and quibbles of the law, may clear his guilty client, but he is not doing the community a good service.

The medical man is prevented, by the rules of the profession, from advertising at all. He may put his card and location in the papers, and he may put on his sign "surgeon," "oculist" or "aurist," but further than that he must not go ; if he does, he is expelled from the profession ; so that those who commence to advertise usually go the full extent. They do not hesitate to promise the most certain cures in all cases that come to them ; they have a specific for every disease, denouncing others around them as quacks, while they are the greatest quacks themselves. If they are successful in deceiving the community, and make money, they are tolerated in society ; and if they can give parties, they will always find enough people to attend them. But they cannot remove the impression from the honest portion of the community that their course has been a deceiving one.

The Dentists are classed with the Physicians ; to advertise is considered derogatory to their standing in the profession, and when they resort to those notorious advertisements which we continually see in the public papers, they place themselves in a position that no respectable person envies. No one wishes to associate with a notoriously bad person. Those who commit acts which the laws punish are afterwards marked by society, and are spurned; but those who escape the law are many times the most culpable; for the person who will deliberately and systematically deceive and swindle, is worse than one who steals from necessity. It is not the fact of advertising that should degrade the person. Dentists all issue cards, or, when they open an office or remove to other localities, either publish or send notices to their patients and others, announcing these changes. It is only when they condescend to the class of advertisements which we see every day in the public papers, that they are to be condemned; for, as we have stated, those who will deceive to obtain practice, will not hesitate to deceive in their practice."

